# Dynamin1 concentration in the prefrontal cortex is associated with cognitive impairment in Lewy body dementia

**DOI:** 10.12688/f1000research.3786.1

**Published:** 2014-05-13

**Authors:** Julie Vallortigara, Sindhoo Rangarajan, David Whitfield, Amani Alghamdi, David Howlett, Tibor Hortobágyi, Mary Johnson, Johannes Attems, Clive Ballard, Alan Thomas, John O’Brien, Dag Aarsland, Paul Francis

**Affiliations:** 1 Wolfson Centre for Age-Related Diseases, King's College London, London, SE1 1UL, UK; 2 Department of Neuropathology, Institute of Pathology, University of Debrecen, Debrecen, H-4032, Hungary; 3 Institute for Ageing and Health, Newcastle University, Newcastle upon Tyne, NE4 5PL, UK; 4 Department of Psychiatry, Cambridge Biomedical Campus, University of Cambridge, Cambridge, CB2 0SP, UK; 5 Department of Neurobiology, Ward Sciences and Society, Karolinska Institute, Stockholm, SE-141, Sweden

**Keywords:** Alzheimer’s disease, Dementia with Lewy bodies, Parkinson’s disease with dementia, synaptic dysfunction, vesicle recycling, synaptic plasticity, beta amyloid, tau, cognitive impairment

## Abstract

Dementia with Lewy Bodies (DLB) and Parkinson’s Disease Dementia (PDD) together, represent the second most common cause of dementia, after Alzheimer’s disease (AD). The synaptic dysfunctions underlying the cognitive decline and psychiatric symptoms observed throughout the development of PDD and DLB are still under investigation. In this study we examined the expression level of Dynamin1 and phospho-CaMKII, key proteins of endocytosis and synaptic plasticity respectively, as potential markers of molecular processes specifically deregulated with DLB and/or PDD. In order to measure the levels of these proteins, we isolated grey matter from post-mortem prefrontal cortex area (BA9), anterior cingulated gyrus (BA24) and parietal cortex (BA40) from DLB and PDD patients in comparison to age-matched controls and a group of AD cases. Clinical and pathological data available included the MMSE score, neuropsychiatric history, and semi-quantitative scores for AD pathology (plaques - tangles) and for α-synuclein (Lewy bodies).

Changes in the expression of the synaptic markers, and correlates with neuropathological features and cognitive decline were predominantly found in the prefrontal cortex. On one hand, levels of Dynamin1 were significantly reduced, and correlated with a higher rate of cognitive decline observed in cases from three dementia groups. On the other hand, the fraction of phospho-CaMKII was decreased, and correlated with a high score of plaques and tangles in BA9. Interestingly, the correlation between the rate of cognitive decline and the level of Dynamin1 remained when the analysis was restricted to the PDD and DLB cases, highlighting an association of Dynamin1 with cognitive decline in people with Lewy Body dementia.

## Introduction

Dementia with Lewy Bodies (DLB) and Parkinson’s disease dementia (PDD) are together the second most common cause of dementia after Alzheimer’s disease (AD) and account for 15–25% of dementias ^1^. They are both characterised by progressive cognitive decline, visual hallucinations and Parkinsonism ^2, 3^. Lewy body dementias (including DLB and PDD) are neuropathologically defined by insoluble α-synuclein aggregates in neuronal somata, forming Lewy bodies and Lewy neurites in neuronal processes ^2^. However, it has been widely reported that Alzheimer-type pathology (most often amyloid plaques, but also to lesser extent neurofibrillary tangles) often coexists with Lewy body pathology ^4– 6^. When compared to the large-scale cortical atrophy of AD and the dopaminergic neuronal cell loss of PD, cortical cell loss in DLB and PDD is less extensive ^7^, suggesting that alternative mechanisms may be responsible for the symptoms associated with DLB and PDD. In addition to Lewy bodies and Lewy neurites, small aggregates of α-synuclein have been identified pre-synaptically in DLB and PDD, raising the possibility of a deleterious effect on synaptic function ^8, 9^. Alongside this, interactions between α-synuclein and synaptic vesicle (SV) recycling proteins have previously been described ^10^, suggesting that SV proteins are affected by the alterations in concentration of pre-synaptic α-synuclein ^11^. It has also been shown that dysfunctional vesicle regulation can lead to dementia-like cognitive deficits ^12^.

In AD there is substantial synaptic loss, which was revealed to provide a better indicator for cognitive impairment than the classical AD morphological changes ^13^. Although there is emerging evidence of synaptic pathology in PD ^14^, much less is known regarding the molecular basis and clinical consequences of the synaptic pathology associated with PD and DLB compared to AD, which may provide an opportunity for therapeutic intervention, through restoration of neurotransmitter tone.

Dynamin1 is a ~ 100kDa protein with GTPase activity, that is involved in many intracellular trafficking processes including SV recycling, neurotransmitter reuptake and receptor internalization ^15, 16^. A considerable body of evidence supports a key role in synaptic transmission. For example, Dynamin1 interacts with other endocytotic proteins including amphiphysin, endophilin and syndapin through its C-terminal proline rich domain (PRD), and as a consequence, pharmacological inhibition leads to disturbance of normal SV- and endosome formation ^17^. Furthermore, Dynamin1 knockout mice demonstrate defects in SV endocytosis during strong, but not mild, neuronal activity ^18^. In addition, Dynamin1 may play a key role in establishing and maintaining mature neuronal structure ^19, 20^. For example, dynamin1 is upregulated during new neurite formation ^21^ and is down-regulated during neurite retraction, and furthermore, silencing the initiation codon for dynamin significantly hampers the formation of axon-like structures.

Ca ^2+^/calmodulin-dependent protein kinase II-α (CaMKII) is a protein kinase highly concentrated in the brain and implicated in synaptic plasticity mechanisms ^22– 24^. Synaptic activity-triggered Ca ^2+^ influx through NMDA receptor channels can activate CaMKII and promote its autophosphorylation at Thr286, which results in a persistently active form of the kinase ^25, 26^. The resulting CaMKII activation is likely to occur at both pre-and postsynaptic sites. At the presynaptic site, CaMKII-mediated phosphorylation of synapsin1 promotes its dissociation from synaptic vesicles, causing increased neurotransmitter release ^27^. Activation of a calcineurin-dependent phosphatase pathway, however, can dephosphorylate CaMKII and reduce its activity ^28^. Interestingly, Calcineurin, which is also activated by calcium, can dephosphorylate Dynamin1 and therefore regulates the SV retrieval processes ^16^.

The synaptic dysfunctions underlying the cognitive decline and psychiatric symptoms in DLB and PDD are still poorly understood. The relative lack of frank neurodegeneration in DLB and PDD combined with the potential importance of synaptic pathology, together with the key role Dynamin1 and CaMKII play in synaptic neurotransmission led us to propose the hypothesis that dysfunctional synaptic plasticity and disrupted vesicle recycling may contribute to cognitive decline. We therefore investigated the concentrations of these two key proteins in prefrontal cortex, anterior cingulate, and parietal cortex regions of DLB and PDD in comparison to controls and AD in relation with cognitive decline as assessed by serial measurements of the Mini-Mental State Examination (MMSE) together with semi-quantitative assessments of plaques, tangles and Lewy bodies within those regions.

## Materials and methods

### Participants, diagnosis and clinical assessment

Post-mortem brain tissue was obtained from several sources; University Hospital Stavanger (Norway), the MRC London Neurodegenerative Diseases Brain Bank, the Thomas Willis Oxford Brain Collection and the Newcastle Brain Tissue Resource. The UK brain banks are part of the Brains for Dementia Research Network. All participants gave informed consent for their tissue to be used in research and the study had ethics approval from the National Research Ethics Service (08/H1010/4). Neuropathological assessment was performed according to standardised neuropathological scoring/grading systems, including Braak staging, Consortium to Establish a Registry for Alzheimer’s Disease (CERAD) scores, Newcastle/McKeith Criteria for Lewy body disease, National Institute on Aging - Alzheimer’s Association (NIA-AA) guidelines and phases of amyloid-β (Aβ) deposition (Aβ-phases) ^2, 29– 32^. Controls were cognitively normal, with only mild age-associated neuropathological changes (e.g., neurofibrillary tangle Braak stage <II) and no history of neurological or psychiatric disease.

Patients were followed prospectively with annual assessments including standardized instruments of cognitive, motor and neuropsychiatric symptoms. Cognitive impairment data consisted of the last Mini-Mental State Examination (MMSE) scores a maximum of two years prior to death ^33^. Final diagnoses for patients are clinico-pathological consensus diagnoses incorporating the one-year rule to differentiate DLB and PDD ^2^. Table 1 shows the demographic details of the patients and controls. Biochemical and histopathological analysis was undertaken on prefrontal cortex (Brodmann area, BA9), anterior cingulate gyrus (BA24) and parietal cortex (BA40). BA9 was selected due to its proposed role in executive function and cognition ^34^, decline of which is a cardinal symptom of DLB and PDD, BA24 was selected for the early development of pathology encountered in this region in DLB and PDD ^35^ whilst BA40 was selected because of its pathological predominance in AD as opposed to DLB and PDD ^36^.

**Table 1.** Summary of subject demographics. Values are mean ± SEM. DLB: Dementia with Lewy Body; PDD: Parkinson’s Disease Dementia; AD: Alzheimer’s Disease. PMD: post mortem delay. Age at death, PMD and pH are mean values, MMSE is the median score prior to death with range in brackets.

	Control	DLB	PDD	AD
Number of cases	25	55	34	16
Age of death (years)	79.8 ± 1.5	81.7 ± 0.9	79.9 ± 1.0	88.0 ± 2.0
PMD (hours)	39.1 ± 4.6	41.3 ± 3.8	33.5 ± 2.7	34.9 ± 6.0
Gender M/F (%)	60/40	56/44	53/47	31/69
Brain pH	6.47 ± 0.07	6.37 ± 0.06	6.44 ± 0.06	6.30 ± 0.08
MMSE (last assessment)	n/a	13 (0–30)	13 (0–27)	10.5 (0–19)

### Immunohistochemistry

Semi-quantitative assessments of Aβ, tau and α-synuclein pathology were conducted blind to clinical diagnosis, by neuropathologists, using a scale of 0 (none), 1 (sparse), 2 (mild) and 3 (severe/frequent) to score sections from BA9, BA24 and BA40 according to published criteria ^35, 36^. For detection of senile Aβ plaques sections were stained with an anti-Aβ 1E8 (gift from GSK) or 4G8 antibody (Covance SIG39220 mouse monoclonal) raised to Aβ17-24, at 1:1000. Tau immunohistochemistry (AT8 antibody (Innogenetics) at 1:200) and silver impregnation (Gallyas or modified Bielschowsky) were used to detect neurofibrillary tangles, neuritic plaques, dystrophic neurites and neuropil threads. α-Synuclein pathology was detected using NCL-SYN antibody (Novacastra Laboratories) at 1:200.

### Preparation of tissue samples for western blotting

Preparation of tissue for western blotting was as previously described ^37^. Briefly, 500mg of frozen tissue was taken from each brain region. Meninges, white matter, blood vessels and clots were dissected from the frozen tissue to leave approximately 200mg of grey matter which was homogenised in ice cold buffer containing 50mM Tris-HCL, 5mM EGTA, 10mM EDTA, ‘complete protease inhibitor cocktail tablets’ (Roche, 1 tablet per 50ml of buffer), and 2μg/ml pepstatin A dissolved in ethanol:DMSO 2:1 (Sigma). Buffer was used at a ratio of 2ml to every 100mg of tissue and homogenisation performed using an IKA Ultra-Turrax mechanical probe (KIA Werke, Germany) until the liquid appeared homogenous.

Protein concentration was established using the Coomassie (Bradford) Protein Assay Kit (Thermo Scientific), briefly 10μl of crude homogenate was diluted 1:50 and read in triplicate at 595nm using a FlexStation 3 (Molecular Devices). Concentration was calculated using a BSA standard curve run at the same time as samples.

### Western blotting

Crude brain homogenate was diluted 4:5 with 5× sample buffer (Genscript MB01015), boiled for 5 minutes then stored at -20°C. Samples were loaded at 20μg/ml total protein on 10% SDS-polyacrylamide gel for protein separation, transferred to nitrocellulose membrane (Hydrobond-C, Amersham) and probed with either anti-totalCaMKIIα (Santa Cruz sc-136212, 1:10000) or anti-phospho-CaMKII (Santa Cruz sc-12886-R, 1:200) and the relevant secondary antibody (IRDye from LI-COR, anti-mouse for total CaMKIIα and anti-rabbit for phospho-CaMKII). Bands were detected using an Odyssey infrared fluorescent scanner, the integral of intensity quantified using Odyssey infrared imaging systems application software version 3.0.16 and expressed as ratios to rat cortex in arbitrary units.

### ELISA

Using the crude brain homogenates already prepared, tissue debris was then removed by centrifugation maintained at 15000× g for 15min at 4°C. The resultant supernatant was collected and the protein concentrations were determined using the Coomassie (Bradford) Protein Assay Kit as previously described. Dynamin1 concentration was measured using a commercial ELISA kit developed by USCNLIFE TM (Wuhan China). The microtiter plate was pre-coated with biotinylated polyclonal antibody specific to Dynamin1. 100μl standards or samples (10 × diluted) were added and incubated for two hours at 37°C. 100μl detection reagent A (avidin conjugated to Horseradish Peroxidase (HRP)) was added to each micro plate well and incubated for 1hr at 37°C. After washing 4 times, 100μl of a TMB (3,3’,5,5’ tetramethyl-benzidine) substrate solution was added to each well and incubated for 1hr at 37°C. Colour developed in proportion to the amount of bound analyte. Finally, the enzyme-substrate reaction was terminated by adding 1N sulphuric acid. Quantification of Dynamin1 was achieved measuring colour changes using a spectrophotometer at a wavelength of 450nm. The concentration of Dynamin1 in the samples was determined by comparing the optical density (OD) of the samples to the standard curve. Within-assay precision for a replicated sample on the same plate (%CV of intra-assay variation) for a selected sample was < 6%. The inter-assay variability, (for same sample analyzed on three different plates) was < 15%.

### Statistical analysis

The normality of the data for each protein was determined using the Shapiro-Wilk test and normalised where necessary. In each case, the protein values were subsequently expressed as residuals (unstandardised) created from the multivariable regression analysis, to eliminate the confounding effect of the demographic variables (gender, post mortem delay (PMD), age at death, length of brain storage) on the protein values. Unstandardised residuals were used in all subsequent analyses. We tested for differences in protein levels between groups using one-way ANOVA and Bonferroni post-hoc test or Kruskall-Wallis ANOVA followed by Mann Whitney *U* test as appropriate Intercorrelations of neurochemical variable and correlations with demographic and clinical features were examined using Pearson product moment ( *r*) or Spearman rank ( *Rs*) correlation as appropriate. Statistical analyses were conducted using SPSS version 20.

## Results

Demographics of the cohort used are summarised in Table 1. AD patients were significantly older at death (one-way ANOVA F(3;126)=6.044, p=0.001) than controls (p=0.001) or patients with DLB (p=0.008) or PDD (p=0.001). There were no significant differences in PMD, tissue pH or gender between diagnostic groups.

### Changes in expression of synaptic proteins with diagnosis

**Figure 1.**
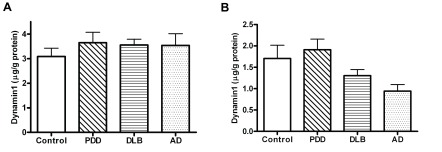
Concentration of Dynamin1 in BA9 and BA24 tissues by ELISA from subjects with PDD, DLB, AD and controls. ( **A**) BA9, ( **B**) BA24, PDD: Parkinson’s Disease Dementia; DLB: Dementia with Lewy Body; AD: Alzheimer’s Disease. Bars represent mean and error bars SEM.

**Figure 2.**
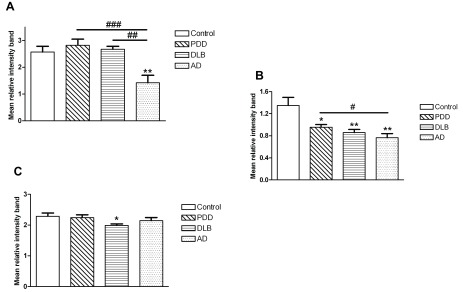
Fraction of phophoCaMKII levels measured by Western blot in BA9, BA24 and BA40 tissues from subjects with PDD, DLB, AD and controls. ( **A**) BA9, ( **B**) BA24 and ( **C**) BA40. Bars represent mean of ratio phospho/totalCaMKII and error bars SEM. ** (p<0.05) and ** (p<0.01) compared to controls, # (p<0.05), ## (p<0.01) and ### (p<0.001) between dementia groups.

The results for protein expression in the prefrontal cortex, anterior cingulate and parietal cortex are shown in Figure 1, Figure 2, and in Table 2. Dynamin1 protein levels were not significantly different between groups (Kruskall-Wallis p=0.682 for BA9; p=0.120 for BA24, Figure 1 and dataset 3 for BA40). On the other hand, changes were found for the expression of CaMKII and the fraction of activated form, i.e. phospho-CaMKII ( Table 2). Indeed, the phospho-/total CaMKII ratio was significantly lower in the AD group compared to control, PDD and DLB groups in the prefrontal cortex (one-way ANOVA F(3,115)=7.129, p<0.001; Figure 2). In the anterior cingulate cortex, this ratio was decreased in all three dementia groups compared to controls (Kruskall-Wallis χ ^2^(3)=14.44, p=0.003; Figure 2). In the same brain region, although the level of Dynamin1 was decreased in the AD group, there was no significant difference between groups. In the parietal cortex, expression of phospho-CaMKII was significantly lower in PDD, DLB and AD compared to controls, with a stronger decrease in the AD group (Kruskall-Wallis χ ^2^(3)=35.942, p<0.001; Table 2). The level of the ratio phospho-/total CaMKII in the DLB group was decreased compared to other groups (one-way ANOVA F(3,114)=3.45, p=0.019; Figure 2).

**Table 2.** Total and phospho-CaMKII levels measured by Western blot in BA9, BA24 and BA40 tissues from subjects with PDD, DLB, AD and controls. Values represent the means of relative intensity band measurements ± SEM. Number in brackets is the number of cases. * (p<0.05), ** (p<0.01) and *** (p<0.001) compared to controls.

	BA9		BA24		BA40	
proteins	TotalCaMKII	pCaMKII	TotalCaMKII	pCaMKII	TotalCaMKII	pCaMKII
control	1.02 ± 0.07 (24)	3.00 ± 0.25 (24)	0.63 ± 0.05 (23)	0.75 ± 0.06 (23)	0.85 ± 0.01 (24)	1.86 ± 0.03 (24)
PDD	0.68 ± 0.05 (32) ***	1.95 ± 0.13 (32) **	0.77 ± 0.03 (33) ***	0.73 ± 0.05 (33)	0.85 ± 0.01 (29)	1.79 ± 0.01 (30) **
DLB	0.98 ± 0.03 (50)	2.89 ± 0.13 (50)	0.90 ± 0.03 (47)	0.80 ± 0.04 (48)	0.85 ± 0.01 (48)	1.76 ± 0.02 (49) **
AD	0.81 ± 0.05 (16)	1.63 ± 0.27 (16)	0.84 ± 0.03 (16)	0.60 ± 0.04 (16)	0.84 ± 0.01 (16)	1.63 ± 0.01 (16)

### Correlates between expression of synaptic proteins and neuropathological features

**Figure 3.**
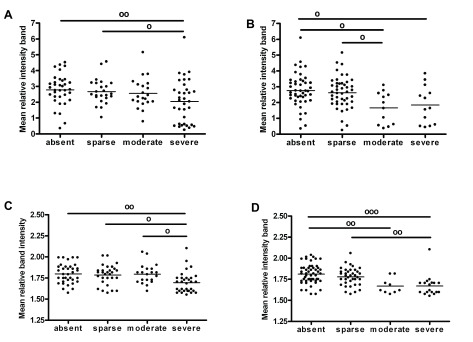
Correlates between synaptic markers and AD neuropathological features. ( **A**) ratio phospho/totalCaMKII level with plaques scores in BA9, ( **B**) ratio phospho/totalCaMKII with plaques scores in BA9, ( **C**) phosphoCaMKII with plaques scores in BA40 and ( **D**) phosphoCaMKII with tangles scores in BA40. Scatter plots represent values and bars the mean for each group. º (p<0.05), ºº (p<0.01) and ººº (p<0.001).

Figure 3 summarises the relationships found between synaptic markers and semi-quantitative scores of AD pathology in BA9 and BA40. The ratio phospho-/total CaMKII was decreased, with a high score of plaques (Kruskall-Wallis χ ^2^(3)=8.549, p=0.036), and medium and high scores of tangles (one-way ANOVA F(3,111)=5.375, p=0.002) in BA9. On the other hand, phospho-CaMKII was significantly decreased with high scores of plaques and tangles in BA40 (one-way ANOVA F(3,111)=5.227, p=0.002 for plaques; one-way ANOVA F(3,112)=9.282, p<0.001 for tangles). There was no correlation between Dynamin1 concentration and neuropathological scores in BA9 or BA40 (one-way ANOVA, p>0.05, see data sets). No significant relationships were found between any neurochemical variable and pathological features in the anterior cingulate cortex (one-way ANOVA, p>0.05, see data sets).

### Synaptic markers expression are decreased with cognitive decline

**Figure 4.**
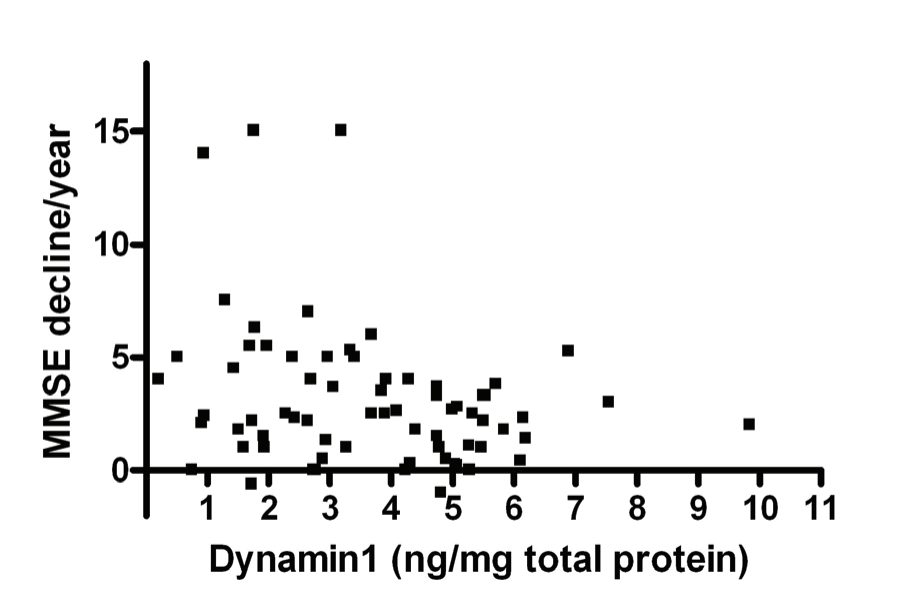
Correlates between Dynamin1 level in BA9 and rate of cognitive decline. Correlation between values of Dynamin1 level in BA9 in dementia cases and the MMSE decline per year.

A significant association between MMSE decline per year and the level of Dynamin1 was observed in BA9, with a decrease in Dynamin1 level with the rate of cognitive decline ( *r*=-0.280, p=0.019, n=70; Figure 4). This correlation is also significant when the analysis was restricted to the PDD and DLB cases ( *r*=-0.327, p=0.014, n=56). In the same brain region, the ratio phospho/totalCaMKII was positively correlated to the MMSE scores before death ( *r*=0.256, p=0.024, n=78), highlighting a decrease in the fraction of activated CaMKII protein with the cognitive deficit. However, when the AD group was excluded from the analysis, there was no correlation between phospho-CaMKII and MMSE.

Data for synaptic proteins expression levels and pathological scores in prefrontal cortex, anterior cingulate gyrus and parietal cortex Dataset1: The dataset includes the values of Dynamin1 and phospho-/total CaMKII measured in the prefrontal cortex, used for the statistic analysis. The first three columns report the semi-quantitative scoring of AD and Lewy Body pathology in this brain region.
Dataset2: The dataset includes the values of Dynamin1 and phospho-/total CaMKII measured in the anterior cingulated gyrus, used for the statistic analysis. The first three columns report the semi-quantitative scoring of AD and Lewy Body pathology in this brain region.
Dataset3: The dataset includes the values of Dynamin1 and phospho-/total CaMKII measured in the parietal cortex, used for the statistic analysis. The first three columns report the semi-quantitative scoring of AD and Lewy Body pathology in this brain region.

## Discussion

The main findings of this study are that the Dynamin1 level in prefrontal cortex, while unaltered between diagnostic groups, was related to the rate of cognitive decline observed in our cohort of people with Lewy body dementia (DLB and PDD) and AD; this relationship remained in the cohort of LBD cases only (when the AD group was excluded from the analysis). On the other hand, altered p(Thr286)CaMKII levels in AD, PDD and DLB parietal cortex, and in AD prefrontal cortex, were associated with high scores of plaques and tangles.

Previous studies have identified decreased expression of Dynamin1 in AD cases, showing particularly a degradation of Dynamin1 at early phase of AD ^38– 40^. A decrease in Dynamin1 expression could create defects in synaptic vesicle recycling, neurotransmitter reuptake and receptor endocytosis ^18^. This would lead to a diminished ability of the neuron to regulate synaptic transmission and may prove to be an example of one of the processes that initiate synaptic dysfunction in dementia. However, Dynamin1 has not previously been implicated in LBD. In our cohort, there was no significant difference in the level of Dynamin1 between diagnosis groups, neither was there a correlation between Dynamin1 and neuropathological features. This is particularly surprising for AD pathology, as some potential links between Dynamin1 expression and Aβ have been reported ^41, 42^. However, these differences could relate to the relatively small comparison group used for this study. Nevertheless the progressive decrease in Dynamin1 in BA9 with the rate of MMSE decline provides new evidence for a role of this protein in cognitive dysfunction in DLB and PDD.

The essential role of CaMKII in long-term synaptic plasticity and cognitive function is well documented ^22– 24^. Here we confirmed this finding with a decreased level of phospho-/total CaMKII, specifically in prefrontal cortex of the AD group. In addition, the level was significantly lower in the prefrontal cortex of dementia patients with a high score of plaques. Moreover, a lower expression of p(Thr286)CaMKII was observed in parietal cortex of patients with a high score of plaques. An interesting finding in another study was the co-localization of CaMKII-α with senile plaques (SPs) ^43^. With respect to the AD-related neuropathology, *in vitro* experiments indicated that CaMKII might participate in the tau protein phosphorylation ^44, 45^. Simonian *et al.* ^46^ found that the majority of tangle-bearing neurons in AD brain expressed CaMKII. We also found an association between the level of p(Thr286)CaMKII and phosphoTau, with a decrease in p/totalCaMKII in prefrontal cortex and phospho-CaMKII in parietal cortex of dementia patients with a moderate or severe spread of plaques. However, when the AD group is excluded from the analysis, there were no correlations between phospho-CaMKII and MMSE or pathological features in any brain region. This is a key finding, as it may rule out the potential role of this kinase in molecular mechanisms leading to the development of cognitive decline in Lewy body dementia. However, it does appear to confirm previous reports of impaired phosphorylation CaMKII in AD ^47, 48^.

Taken together, previous studies and the findings reported here suggest that Ca ^2+^ dysregulation in AD, and LBD within selected brain regions, may be sufficient to initiate a deregulation in CaMKII- and Dynamin1-dependent molecular pathways, and that this, in turn, may contribute to cognitive decline.

## Data availability

*figshare*: Data for synaptic proteins expression levels and pathological scores in prefrontal cortex, anterior cingulate gyrus and parietal cortex, http://dx.doi.org/10.6084/m9.figshare.987087 ^49^
