# Supplementary material for: Dynamin1 concentration in the prefrontal cortex is associated with cognitive impairment in Lewy body dementia
Source: F1000Res. 2014 May 13;3:108. [Version 1] doi: 10.12688/f1000research.3786.1 (PMC4309165; doi:10.12688/f1000research.3786.1)
Supplement: Peer review history

# Peer review: Referee response for version 1

**DOI:** 10.5256/f1000research.4055.r7429

**Published:** 2015-01-23

**Authors:** Tim Anderson

**Affiliations:** 1 New Zealand Brain Research Institute, University of Otago, Christchurch, New Zealand

There remains uncertainty regarding the relative contributions of alpha-synuclein, tau and amyloid pathology to the cognitive impairment in the Lewy body dementias (LBDs) - Parkinson’s disease dementia (PDD) and Dementia with Lewy bodies (DLB). On the justifiable preposition that whatever the underling pathology, synaptic dysfunction is a key causative mechanism of cognitive change in PDD and DLB (as it is in Alzheimer’s disease, AD), the authors in this excellent study on postmortem brain tissue examined expression levels and ratios of two synaptic proteins, Dynamin1 and CaMKII, in PDD and DLB in comparison with controls and AD, and correlated them with rate of MMSE decline. The key findings were firstly, that Dynamin1 levels in prefrontal cortex were correlated with rate of cognitive (MMSE) decline in all three dementia groups despite mean concentrations being no different from controls, and secondly, that CaMKII levels in parietal cortex were correlated with degree of plaque and tangle formation. Thus, the authors reasonably conclude that that (calcium-related) synaptic protein pathways are altered in the LBDs and likely contribute to cognitive impairment.

Specific comments:

1. The authors chose cortical regions according to known affectation in the three dementias. It might have been of interest to have included the striatum as well given that striatal amyloid changes have been reported in the LBDs.
2. The AD group comprised greater than 2:1 females to males, whereas the control and LBD groups comprised more males than females. Is there (and was there) a gender difference in the levels of the two synaptic proteins examined? If so, could that have had any influence on the results?
3. There is inconsistency in the concentration units of Dynamin1 in figures 1 and 4 (μgm/g protein and ng/g protein respectively).
4. Figure 4 depicts the significant relationship between Dynamin1 levels and MMSE progression. Three patients had a decline of around 15 MMSE points per year, a remarkably rapid progression and considerably greater than the remainder of the dementia patients. Is there something different about these patients and does the significant relationship still hold if these three are omitted from the analysis?

The association of Dynamin1 levels with rate of cognitive decline in the face of normal Dynamin1 levels seems surprising and a clearer explanation of this apparent ambiguity would be welcome.

I have read this submission. I believe that I have an appropriate level of expertise to confirm that it is of an acceptable scientific standard.

# Peer review: Referee response for version 1

**DOI:** 10.5256/f1000research.4055.r5607

**Published:** 2014-09-30

**Authors:** Stephen M. Gentleman

**Affiliations:** 1 Department of Medicine, Imperial College London, London, UK

This is well -presented study reporting an association between cognitive change and levels of the synaptic protein Dynamin1 in post-mortem tissue from the prefrontal cortex of Lewy body dementia patients. There is ongoing debate in the literature as to the respective roles of alpha synuclein, A-beta peptide and tau pathologies in the pathogenesis of Lewy body dementia so it is interesting to see a change of focus to the presumed anatomical basis of breakdown in inter-neuronal communication i.e. synapse loss or dysfunction.

The title is appropriate, the abstract provides a fair summary of the study and the methodology is described in sufficient detail. The discussion is well written and the conclusion regarding possible links between deregulation of synaptic proteins and cognitive decline is compelling. Just a couple of comments:

- In this study DLB and PDD are grouped together under the heading of Lewy body dementias. For those outside the field it would perhaps be useful to provide more detail on the clinical and  neuropathological (?) differences between the two conditions.
- A simple diagram showing the possible functional inter-relationship between Dynamin1 and CaMKII might be useful to help justify the choice of these two markers to look at synaptic function.

**Minor**

In the abstract "cingulated" needs changing to cingulate

I have read this submission. I believe that I have an appropriate level of expertise to confirm that it is of an acceptable scientific standard.
